# Seasonal expression of reproductive axis-related neuroendocrine genes and their relation with ovarian maturation in captive yellowtail kingfish (*Seriola lalandi*)

**DOI:** 10.1186/s40659-025-00622-5

**Published:** 2025-08-08

**Authors:** Jaime Palomino, Ayleen Olea, Stevanie Ramírez, Phillip Dettleff, Ricardo D. Moreno

**Affiliations:** 1 Escuela de Medicina Veterinaria, Centro de Estudios e Investigación en Salud y Sociedad (CEISS), Facultad de Ciencias Médicas, Universidad Bernardo O’Higgins, Casilla 2 Correo 15, Santiago, Chile; 2 Laboratorio de Reproducción Animal, Facultad de Ciencias Veterinarias y Pecuarias, Universidad de Chile, Santiago, Chile; 3 Escuela de Medicina Veterinaria, Facultad de Agronomía y Sistemas Naturales, Facultad de Ciencias Biológicas y Facultad de Medicina, Pontificia Universidad Católica de Chile, Santiago, Chile; 4 Facultad de Ciencias Biológicas, Pontificia Universidad Católica de Chile, Santiago, Chile

## Abstract

The yellowtail kingfish (*Seriola lalandi*) is a key species for the diversification of Chilean aquaculture. While controlled reproduction is essential for reliable fish production, the physiological and molecular bases underlying its reproductive cycle remain insufficiently explored. This study aimed to assess the expression patterns of brain-expressed neuroendocrine mRNAs involved in the activation of brain-pituitary-gonad (BPG) axis throughout different year seasons and to correlate them with ovarian maturation stages in *S. lalandi* females under captive conditions. Reproductive stages were determined by ovarian histology and gonadosomatic index (GSI) analysis. Expression levels of GnRH1 and GnRH2 genes (*gnrh1* and *gnrh2*) as well as melatonin (*mtn1ra*), dopamine (*drd2a*), and kisspeptin (*kiss1r*) receptors were quantified by real time PCR in brain samples from adult individuals. Fish were maintained in temperature- and photoperiod-controlled tanks simulating the four seasons. Histological and GSI analyses identified four distinct reproductive stages. Gene expression peaked in winter and decreased in autumn, aligning with the seasonal progression of ovarian development. These results are consistent with the expected activation of the reproductive axis during the winter months in *S. lalandi*. Notably, the elevated expression of *drd2a* in winter suggests that modulation of GnRH action on pituitary function may not rely solely on dopaminergic inhibition. Taken together, our findings indicate that *S. lalandi* exhibits reproductive dynamics under captivity that mirror those of wild populations, supporting its use as a reliable model for studying reproductive physiology. Moreover, its responsiveness to environmental cues under controlled conditions enables the development of experimental strategies that would be logistically unfeasible in the wild.

## Introduction

The yellowtail kingfish (*Seriola lalandi*) is a native fish species with a great potential to diversify Chilean aquaculture. However, several technological challenges across its production cycle have hindered proper scaling. Irregular spawning behavior in broodstock and premature gonadal development in juveniles within fattening units are among the most reported issues—problems observed in farmed fish species including Seriola genus [1]. Addressing these challenges requires a deep understanding of species-specific reproductive physiology, in which the characterization of neuroendocrine factors and their receptors within the reproductive axis is a fundamental step.

Available studies in wild *S. lalandi* include gonadal histology and its correlation with plasma steroid levels [2]. This species is classified as a multiple spawner with synchronous oocyte development and a spawning period that occurs during the summer. These traits were later confirmed in a study on captive-conditioned broodstock, which also described mating behavior and early embryonic and larval development [3]. Additionally, in previous work, we assessed expression of key genes for oocyte maturation and quality of both spawned eggs and early embryos [4–6].

Reproductive success in teleosts depends on the coordinated function of the brain–pituitary–gonadal (BPG) axis [7]. Although evolutionarily conserved, the expression patterns of key molecules in this axis vary among species and across reproductive stages, shaping species-specific reproductive strategies. Research in model species—such as zebrafish, salmonids, and some marine fish—has shown that the brain responds to environmental stimuli (primarily photoperiod) by releasing melatonin, which then triggers neurohormones that stimulate the synthesis and release of pituitary gonadotropins, ultimately regulating gametogenesis and steroidogenesis [8]. In females, estradiol stimulates hepatic production of vitellogenin, a precursor protein incorporated into growing oocytes during vitellogenesis [9]. Vitellogenin is processed into yolk proteins and free amino acids for use during embryonic development [10] and its progression can be monitored through ovarian histology [11].

Temporal changes in melatonin receptor expression define the hormone’s effects throughout the reproductive cycle [12–14]. Melatonin stimulates GnRH-secreting neurons in the hypothalamus, and GnRH then binds to its receptor (GnRhr) in pituitary cells to promote gonadotropin release [15]. Teleost expresses two or three forms of GnRH [16]. GnRH1 in the preoptic area, GnRH2 in the midbrain, and GnRH3 in the terminal nerve ganglion [17]. Kisspeptins also play a pivotal role in vertebrate reproduction by promoting GnRH release. While mammals possess a single ligand (*kiss1*) and one receptor (*kiss1r*), teleosts underwent additional genome duplications, giving rise to two ligands (*kiss1* and *kiss2*) and up to four receptor genes [18, 19]. Ligand/receptor presence is species-specific: for example, only *kiss2* and *kiss2r* are found in *Solea senegalensis* [20]. Whereas both *kiss1r* and *kiss2r* are present in *Carassius auratus* and *Morone saxatilis* [21, 22].

Conversely, dopamine (DA) has been proposed as a negative regulator of GnRH in some fish species. DA binds to its receptor (*drd2a*), inhibiting GnRH synthesis and release, downregulating GnRhr expression, and blocking gonadotropin release from the pituitary [23].

Therefore, available data from studies performed in several fish species indicate that the activation of BPG axis depends on the expression profiles of molecules belonging this axis, which is reflected in particular traits of the reproductive cycle and the temporal features of the ovarian development. In *S. lalandi*, the expression profiles and functional roles of molecules related to BPG axis activation throughout the reproductive cycle remain unknown. Thus, to know the temporal expression pattern of neuroendocrine factors involved in the activation of the BPG, is an important step to understand the physiology of reproduction of pelagic fishes in the wild, and to propose new effective farming management procedures. Therefore, the hypothesis of the present work was the seasonal mRNA face patterns of *gnrh* genes and neuroendocrine receptors in adult brain individuals correlates with ovarian maturation stages throughout the year in *Seriola lalandi* females, under captivity.

## Materials and methods

### Ethical approval

All experimental procedures were approved by the Ethics and Animal Care Committees of the participating institutions. The protocols from Universidad Bernardo O’Higgins and Pontificia Universidad Católica de Chile (Protocol ID 190613051) were validated by the Research Ethics Committee of the Chilean National Foundation for Scientific and Technological Research (ANID).

### Sample collection

Brain and ovary samples were obtained from 24 adult females belonging to a captive broodstock maintained by Acuinor S.A., a company located in Caldera, Atacama Region, Chile. The broodstock consisted of approximately 100 individuals, aged 8–15 years, distributed across four 30,000 L indoor tanks at a female-to-male ratio of 1:2. Through photothermal manipulation, each tank was conditioned to simulate seasonal environmental variations occurring throughout the year. Average values representing mid-season conditions are shown in Table 1. To capture distinct stages of the reproductive cycle, total brain samples were collected from nine females during each season. Samples were preserved in RNAlater^®^ Solution (Ambion^®^, Austin, Texas, USA) and stored at − 80 °C for subsequent RT-qPCR analysis. Additionally, to confirm that the gonadal stage of each dissected female was consistent with the simulated environmental season, total body weight and gonad weight were recorded. The gonadosomatic index (GSI) was calculated as follows: GSI = (gonad weight/body weight) × 100 (2) and the ovary was processed to histology.

**Table 1:** Ranges values of temperature, light and darkness that were adopted in each tank at the middle of each season

Season	Temperature ºC	Hours of light	Hours of darkness
Summer	20–22.5	14–15	9–10
Autumn	17.5–19.5	12–13	11–12
Winter	13.5–16	9–10	14–15
Spring	17.5–19	13–14	10–11

### Ovarian histology

Gonadal stages were determined by histological analysis. A fraction of the middle part of the ovarian tissue representing different macroscopic appearance were fixed in Bouin’s solution and processed by an external laboratory using standard paraffin-embedding protocols. Tissue sections were stained with hematoxylin and eosin, and at least three slides per fish were imaged with an inverted microscope (IX71; Olympus). Developmental stages were classified according to previously established morphological criteria [2, 11].

### RNA extraction and cDNA synthesis

Total RNA was extracted from brain tissue using affinity columns from the GeneJET^™^ RNA Purification Kit (Thermo Fisher Scientific, Massachusetts, USA), following the manufacturer’s instructions. RNA concentration and purity were assessed by spectrophotometry at 260 nm using an Epoch microplate spectrophotometer (BioTek, Vermont, USA). RNA samples were stored at − 80 °C until further use. Complementary DNA (cDNA) was synthesized using the AffinityScript qPCR cDNA Synthesis Kit (Agilent Technologies, Santa Clara, USA) and quantified with a fluorometer using the Qubit^®^ ssDNA Assay Kit (Molecular Probes^®^, Invitrogen^™^). cDNA samples were stored at − 20 °C until analysis.

### Primer design and RT-qPCR analysis

The expression of mRNA of gonadotropin releasing hormone 1 and 2 (*gnrh1, gnrh2)*, melatonin 1 (*mtn1ra*), kisspeptin 1 (*kiss1r*) and dopamine receptor (*drd2a*) associated with the brain–pituitary–gonadal (BPG) axis was analyzed by RT-qPCR. Primers were designed using gene sequences for *S. lalandi* available in GenBank and generated with Primer3Plus software under default parameters. Primer specificity and absence of dimers or secondary structures were verified using NetPrimer software. Prior to gene expression analysis, qPCR conditions were standardized using pooled brain cDNA. Optimal primer concentrations and amplification efficiency (E) were determined via standard curves prepared from a six-point, sixfold dilution series (1:1–1:1250). Only primers yielding amplification efficiencies near 100% and correlation coefficients (r) ≥ 0.99 were used. RT-qPCR reactions were performed in triplicate using 10 ng of cDNA per reaction. No-template and no-reverse transcriptase controls were included in each run. Primer sets were accepted if melting curve analyses showed a single peak, consistent with the predicted amplicon size. Amplifications were carried out using a Mic-qPCR thermocycler (Biomolecular Systems, Queensland, Australia) and Maxima SYBR Green qPCR Master Mix (Thermo). Ct values were obtained for each gene and converted into relative expression values (Q) using the ΔCt method [24]. To select suitable reference genes for normalization of gene expression data, a stability analysis was conducted on four housekeeping genes (*actb*, *map1b*, *gapdh*, and *18srrna*) using brain samples collected across different seasons. Gene stability was evaluated using the NormFinder algorithm [25]. The housekeeping genes *actb* and *map1b* exhibited the most stable expression profiles according to the NormFinder analysis and were therefore selected for normalization of relative gene expression data in this study. The primers used for mRNA quantification of neuroendocrine receptors and reference genes are listed in Table 2.

**Table 2:** Sequences of qPCR primers

Gen	Entrez gene ID	Forward sequence (5′−3′)	Reverse sequence (3′−5′)	pb
*mtnr1a*	111,663,243	AAGGTGCAGGAGTAAACCCG	TGCGTCCTTCCTTGTCTCTC	395
*drd2a*	111,665,034	AGTGGAACTGACACTGTGGAA	AGCCAAGGACCTAAGATGGC	245
*gnrh1*	111,645,105	GTCGCATGTTGGGTTGTTCA	GGTTAAACCTCTGGCAGTCTTC	150
*gnrh2*	111,670,995	AGATTAAGCTGTGCGAGGCA	AGGTCTGTGATTACAGTGGGACA	152
*kiss1r*	111,644,634	ATGAGCCTCACTGCTGAAGG	TTCTCCCACTTTGCAGCTCT	279
*actb*	111,225,231	AGGGAAATCGTGCGTGACAT	GCTGAAGTTGTTGGGCGTTT	563
*map1b*	111,647,584	TCATCAAGATTATCAGGAGGCG	GGAAGCATACACCATGTAGAGG	158

### Statistical analysis

Brain and ovary samples were grouped according to season (autumn, winter, spring, and summer). Three pooled brain samples were analyzed for each season, with each pool consisting of three individuals (9 brains total each season). To evaluate if data were normally distributed, a Shapiro–Wilk test was performed. Differences in mRNA expression levels (pooled brain samples) and GSI (three different ovaries from each season) values among seasons were assessed using one-way ANOVA followed by Tukey’s post hoc test [26]. Statistical analyses were performed with InfoStat Professional software (Version 2004; National University of Córdoba, Argentina). Differences were considered statistically significant at *P* < 0.05.

## Results

### Gonadal stage determination

**Fig. 1:**
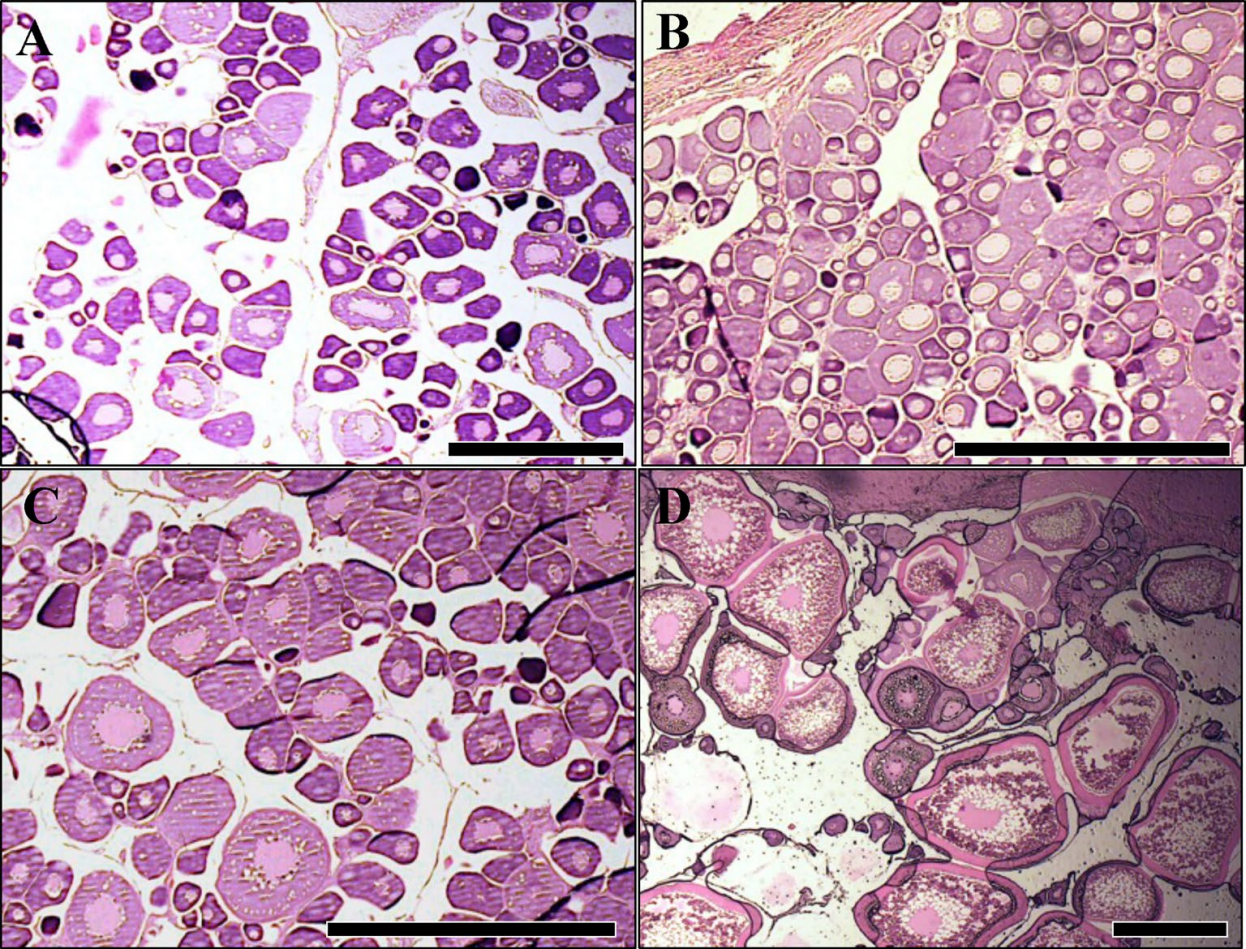
Photomicrographs of hematoxylin–eosin ovarian tissues illustrating different *S. lalandi* reproductive phases. **A:** G1 stage, showing primary growth oocytes (PG), atresia (A), and post-ovulatory follicle complexes (POF). **B:** G2 stage, where primary growth oocytes predominate, with no cortical alveoli or atresia. **C:** G3 stage, representing early vitellogenesis, where oocytes display increased diameter and a layer of cortical alveolar granules (CA) near the nuclear membrane. **D:** G4 stage, indicative of full ovarian activity, showing various vitellogenic stages (Vtg1–Vtg3) and hydrated oocytes (H). Scale bars 100 μm

**Fig. 2:**
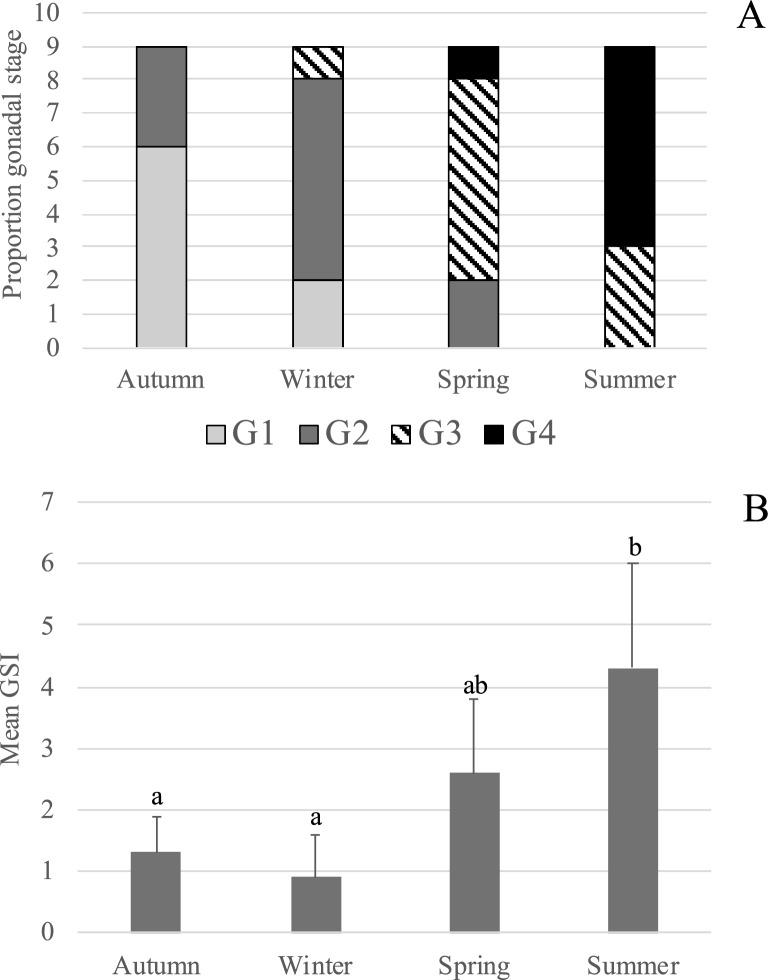
Distribution of ovarian maturation stages and gonadosomatic index (GSI) in adult *S. lalandi* under controlled conditions. **A:** Seasonal distribution of ovarian developmental stages (G1–G4) observed in the nine sampled females at each season. **B:** Seasonal variation in the mean gonadosomatic index (GSI) obtained from three different ovaries from each season (n = 3, a total of 9 ovaries). Different letters indicate statistically significant differences (p < 0.05)

Ovary samples collected from adult females during different seasons revealed four distinct histological stages of ovarian maturation, classified from G1 to G4 (Fig. 1). G1 was a regenerating phase, characterized by the presence of primary growth oocytes (PG), atretic follicles (A), and post-ovulatory follicle complexes (POF) (Fig. 1A). G2 represents a resting phase, in which PG oocytes predominated, and no cortical alveoli or atretic follicles were evident (Fig. 1B). G3 corresponded to an early maturation stage, where vitellogenesis had initiated, and some oocytes had increased in diameter and displayed a layer of cortical alveolar granules near the nuclear membrane (Fig. 1C). Finally, G4 represented an active reproductive phase, with the simultaneous presence of vitellogenic oocytes at various stages (Vtg1–Vtg3) and hydrated oocytes (H), indicative of ongoing spawning (Fig. 1D). In each ovary assessed, the same developmental stage was consistently observed across sections. The seasonal frequency of these ovarian stages is shown in Fig. 2A. Although more than one stage was observed in each season, the most representative stages were G1 in autumn, G2 in winter, G3 in spring, and G4 in summer. GSI values and their seasonal variation are shown in Fig. 2B. GSI values in summer were significantly higher than those in autumn and winter, and comparable to values observed in spring (p < 0.05).

### Expression of neuroendocrine factors mRNA

**Fig. 3:**
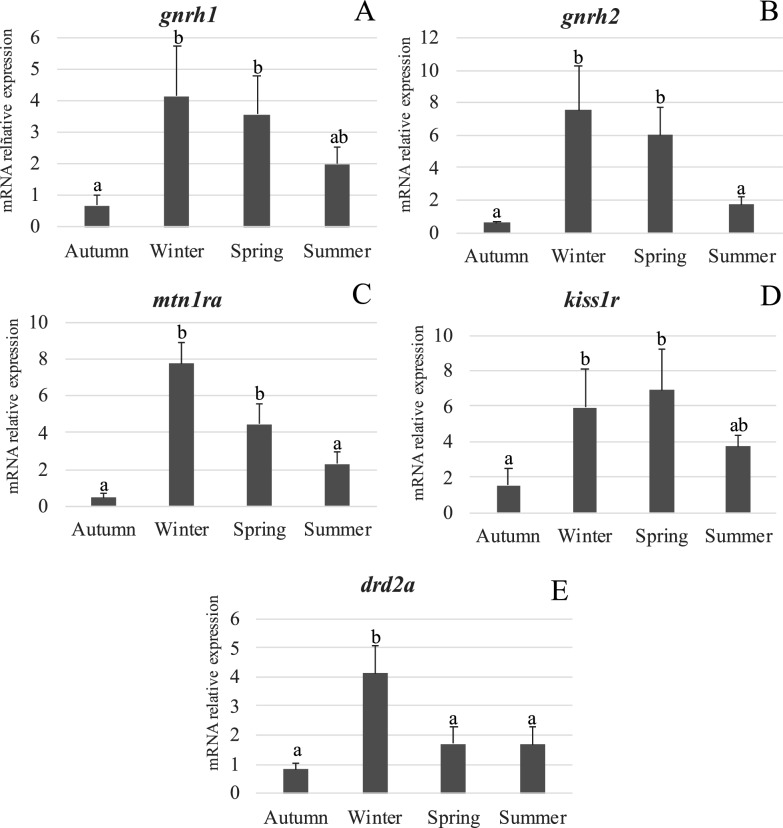
Expression pattern of neuroendocrine genes in *S. lalandi* pooled brain samples collected from captive females throughout different seasons of the year. **A:** *gnrh1*; **B:** *gnrh2*; **C:** *mtnr1a*; **D:** *kiss1r* and **E:** *drd2a*. Different letters indicate significant differences among seasons. Each value represent data obtained from three pooled brain samples, each one consisting of three brains (n = 3, p < 0.05)

Neuroendocrine mRNAs were differentially expressed in brain samples of *S. lalandi* collected throughout the year. GnRH isoforms 1 and 2 (*gnrh1* and *gnrh2*) were detected in all samples. Expression of *gnrh1* increased significantly (p < 0.05) in winter and spring, while it was intermediate in summer and not significantly different from other seasons (Fig. 3A). A similar seasonal pattern was observed for *gnrh2*, with significantly higher expression in winter and spring than in summer and autumn (p < 0.05) (Fig. 3B). For the melatonin receptor gene (*mtn1ra*), expression was significantly higher in winter, and spring compared to autumn and summer, which exhibited the lowest level (p < 0.05) (Fig. 3C). The expression of *kiss1r* was lowest in autumn, with a significant increase in winter and spring. In summer, expression levels did not differ significantly from the other seasons (Fig. 3D). Finally, *drd2a* was expressed in all brain samples, with significantly higher levels in winter compared to the other seasons, which showed no significant differences (p < 0.05) among them (Fig. 3E).

## Discussion

We report for the first time the seasonal expression patterns of neuroendocrine genes and receptor genes associated with the reproductive axis in adult female *S. lalandi* maintained under controlled captive conditions. The expression levels of these genes varied significantly across photoperiod and water temperature changes, suggesting seasonal regulation of the reproductive activity in this species. The ovarian stages identified (G1 to G4) corresponded well with those previously described in teleosts exhibiting similar reproductive strategies [11]. These stages were seasonally distributed, with G1 predominant in autumn and G4 in summer—a distribution that aligned with GSI values and previous findings in wild adult *S. lalandi* collected of the coast of New Zealand [2]. This seasonal association between reproductive parameters and environmental simulation supports the validity of our experimental design and confirms the appropriateness of the brain samples used for molecular analyses. Moreover, our data show that *S. lalandi* maintained in captivity under controlled conditions continues to exhibit seasonal variation in classic reproductive parameters, mimicking patterns observed in the wild.

Melatonin is a well-established regulator of circadian and seasonal physiological rhythms, acting through its receptors to mediate photoperiodic control of reproduction [27]. Among the four melatonin receptors described in fish (*mtnr1a*, *mtnr1b*, *mtnr1c*, and *mtnr1d*), *mtnr1a* was selected for this study due to its broad tissue distribution and its known relevance to reproductive regulation [12, 28]. Our results show that *mtnr1a* expression peaked in winter, coinciding with the period of prolonged darkness where high levels of melatonin are expected. The coincidence of elevated levels melatonin and the *mtnr1a* expression has been also demonstrated in *Solea seleganense* [14, 29], where the highest levels of both components were detected at summer solstice, after the spawning period. So, it seems that melatonin and its receptor levels are coincident with initiating a preparatory phase of the reproductive cycle in *S. lalandi*. These findings are consistent with reports in other teleosts, where photoperiodic cues, detected by both the pineal organ and retina, influence reproductive cyclicity [27, 30].

Consistent with *mtnr1a* expression, transcripts of *gnrh1* and *gnrh2* also increased during winter and declined significantly in summer. This temporal alignment supports the hypothesis that melatonin signaling may enhance GnRH expression as part of the early activation of the reproductive axis. While ligand-receptor affinities for the GnRH isoforms in *S. lalandi* remain uncharacterized, studies in zebrafish, goldfish, and sea bass suggest functional differentiation among receptor subtypes [7, 31]. Notably, ovarian samples collected in winter were mainly in the G2 stage, associated with early vitellogenesis. The upregulation of GnRH mRNAs and neuroendocrine receptors levels, from autumn to winter, may reflect a preparatory phase leading to ovary maturation during spring and summer conditions, where their levels remain elevated (*gnrh1* and *kiss1r*) or gradually decrease (*mtn1ra* and *gnrh2*). Together, these profiles suggest that environmental cues stimulate melatonin signaling, thereby priming the brain’s responsiveness to GnRH.

*Kiss1r* expression was also significantly upregulated in winter and spring. Kisspeptin is known to act upstream of GnRH and plays a central role in regulating seasonal reproduction and pubertal onset in fish [19]. The expression profile observed here suggests that *kiss1r* is involved in early reproductive axis activation prior to full gonadal maturation. While the role of *kiss2r* is better characterized in some specie [32, 33], our findings point to *kiss1r* as part of an anticipatory regulatory mechanism preceding vitellogenesis.

Unexpectedly, *drd2a* expression also peaked in winter, mirroring the patterns of the stimulatory receptor genes. In many teleost species, dopamine acts as a reproductive inhibitor by binding to D2-like receptors and suppressing GnRH secretion [23]. However, *drd2a* expression paralleled that of stimulatory components, raising questions about its potential modulatory role. Alternative explanations could be that the increased expression of this receptor is a compensatory mechanism in response to the decrease in dopamine. Alternatively, since the study used whole brain samples, this increase in *drd2a* could be occurring in areas other than the pituitary. Recent evidence suggests that gonadotropin-inhibitory hormone (GnIH) may be a central inhibitory factor in teleost reproduction [30]. In fact, GnIH treatment has been shown to downregulate GnRH and its receptors in *S. lalandi* [34], supporting the hypothesis that GnIH, rather than dopamine, serves as the principal negative modulator of reproductive activity. The elevated *drd2a* expression observed in winter could reflect a positive compensatory upregulation or the involvement of other inhibitory pathways, such as GnIH.

The coordinated upregulation of *mtnr1a*, *gnrh1*, *gnrh2*, and *kiss1r* suggests a tightly integrated endocrine network anticipating reproductive activation at very early stages of gametogenesis. The parallel increase in *drd2a* expression suggest this preparatory state rather than indicating inhibition. Similar observations have been described in others teleost species such as flatfish [35], European Sea Bass [36] and grey mullets [37]. However, future studies regarding brain localization of dopamine receptors and functional assays using DA antagonist treatment, are required to validate the potential non-inhibitory effect over *S. lalandi* BPG axis. The timing of *kiss1r* upregulation, preceding major ovarian development, suggests a role for kisspeptin in early axis activation rather than in the direct induction of ovulation. Altogether, our results support shows a correlation between neuro-endocrine-related gene expression pattern and ovarian maturation stages in female *Seriola lalandi*, suggesting that controlled captivity condition preserve physiological responses to environmental changes, as observed in wild type species.

## Data Availability

The datasets used and/or analyzed during the current study are available from the corresponding author on reasonable request.
